# Evaluation One Year after DAIR Treatment in 91 Suspected Early Prosthetic Joint Infections in Primary Knee and Hip Arthroplasty

**DOI:** 10.7150/jbji.37757

**Published:** 2019-10-15

**Authors:** Anouk M.E. Jacobs, Lucia J.J. Valkering, Menno Bénard, Jacques F. Meis, Jon H.M. Goosen

**Affiliations:** 1 Department of Orthopaedic Surgery, Prosthetic Joint Infection Unit, Sint Maartenskliniek, Nijmegen, the Netherlands; 2 Sint Maartenskliniek Research, Sint Maartenskliniek, Nijmegen, the Netherlands; 3 Department of Medical Microbiology and Infectious Diseases, Canisius-Wilhelmina Ziekenhuis, Nijmegen, the Netherlands; 4 Department of Medical Microbiology, Radboud University Medical Center, Nijmegen, the Netherlands

**Keywords:** prosthetic joint infection, DAIR, total knee arthroplasty, total hip arthroplasty

## Abstract

Introduction: Early recognition and appropriate initial treatment with debridement, antibiotics and implant retention (DAIR) if a suspicion of an early prosthetic joint infection (PJI) is present can eradicate infection on first attempt and prevent implant failure. We evaluated the outcome after 1 year of patients treated with DAIR after primary total knee arthroplasty (TKA) or total hip arthroplasty (THA). Furthermore, we determined preoperative, microbiology, and treatment factors related to failure after DAIR.

Methods: A retrospective cohort study was assembled with 91 patients undergoing DAIR with a high suspicion of an early PJI. Records were reviewed for demographics, preoperative laboratory results, microbiological data, given treatment and postoperative follow-up. The primary outcome was infection-free implant survival at 1 year. Repeated DAIR was not considered as treatment failure.

Results: The rate of infection-free implant survival following DAIR in a suspected early PJI was 85% (95% confidence intervals (CI) 78-91). Cultures remained negative in 20 patients, with no occurrence of infection during follow-up. A higher failure rate was seen in early PJI caused by* Enterococcus faecalis* (p=0.04)*.* Multivariate analysis showed a statistically significant association between treatment failure and high C-reactive protein level (CRP >100) (odds ratio 10.0, 95% CI [1.5-70]) and multiple DAIR procedures (≥2) (odds ratio 5.0, 95%CI [1.1-23]).

Conclusion: If an early PJI is suspected DAIR is the appointed treatment with up to 2 debridement procedures. Since culture-negative DAIRs were not related to any complications during follow-up, overtreatment of suspected PJI seems to do no significant harm with respect to implant failure.

## Introduction

Prosthetic joint infections (PJI) are the leading cause of revision in total knee arthroplasty (TKA) and the third most common cause of revision in total hip arthroplasty (THA) 1, 2. With a prevalence ranging from 0.3% to 4% in knee and hip arthroplasties it is considered as a serious complication with considerable morbidity and economic burden 3-5. Early recognition and appropriate initial treatment is important to eradicate infection on first attempt and prevent implant failure.

A widely accepted classification distinguishes 3 groups, early (<3 months), delayed/low-grade (3-24 months), and late (>24 months) infections with a proposed treatment algorithm for each group 6. Early infections can be eliminated with secondary prevention, through surgical debridement, antibiotic treatment, and implant retention (DAIR) 7, 8.

State of the art clinical practice recommends DAIR for an early PJI, provided that the prosthesis is stable, the duration of symptoms does not exceed the length of 3 weeks, the skin and soft tissues are intact, and the causative pathogen is susceptible to a biofilm-active agent 8. Previous studies have shown that success rates of DAIR vary widely from 57-89% 9-13. Failure of DAIR is related to preoperative available parameters (patient-related, symptom-related, laboratory parameters), culture/microorganism related factors, and treatment associated data.

Since January 2012 we implemented DAIR for treatment of patients with a high suspicion of an early PJI following the latest guidelines 8.We evaluated the infection-free survival rate of an early PJI 1 year after DAIR. Furthermore, we determined patient, microbiology, and treatment factors related to failure after DAIR.

## Patients and methods

### Patient selection

We retrospectively reviewed all patients who underwent a DAIR procedure between January 2012 and December 2014 within 3 months after the implantation of a primary TKA or THA. Patients were excluded if results of intraoperative cultures were not available or follow-up after primary joint arthroplasty was less than 1 year. Charts were reviewed to obtain details on demographics, duration of wound leakage, signs of an acute infections, preoperative inflammatory markers, microbiological data and postoperative follow-up. Approval of the hospital ethical review committee was obtained.

### Treatment

PJIs were treated by a multidisciplinary team including the orthopedic surgeon, infectious disease physician, and medical microbiologist. In case of a high suspicion of an early PJI the decision for DAIR was made by the treating surgeon, in consultation with the orthopaedic team. A high suspicion of an early PJI was based on a prolonged wound leakage of at least 7 days, symptoms indicating acute inflammation (increase of rubor, calor, dolor, tumor, and/or fever (>38.5ºC)), and/or raised serum inflammatory markers. Serum CRP, ESR and WBC were performed routinely in case of the above mentioned symptoms. A radiograph of the joint was used to exclude other pathology, like a fracture or dislocation. The surgical procedure of DAIR consisted of opening the joint using the incision of the previously used approach. Tissue samples were obtained with separate clean instruments (at least 6) from synovium, capsule, and interfaces. After this, the joint was thoroughly debrided including synovial resection. The exchange of modular components took place in about half of the cases (based on own practice of the operating orthopedic surgeon), but this was not standard procedure. Hereafter, the joint and wound were thoroughly irrigated with 6 liters of saline using the pulse lavage system. The joint capsule, subcutis, and cutis were closed with the use of a wound drain. The standard procedure did not consist of the use of local antibiotics. Antibiotic treatment with cefazolin (1000mg thrice a day intravenously) was started intraoperatively after obtaining the tissue cultures and was continued until the results of tissue cultures were available. When culture results were negative, antibiotic treatment was ceased with a maximum duration of 14 days. If 2 or more tissue cultures were considered positive for the same microorganism the antibiotic treatment was continued for 3 months. The type of agent was adjusted based on the susceptibility data of the known microorganism. For polymicrobial infections an antibiotic regimen with activity against all cultured pathogens was used. DAIR was repeated if clinical symptoms and laboratory signs did not improve within 10 days of the previous debridement. A maximum of 3 to 4 debridements were performed in some patients. If the infection did not resolve the decision to remove the implant was made by the surgeon in consultation with the multidisciplinary team.

### Microbiological methods

Intraoperative periprosthetic tissue cultures were routinely obtained in an aseptic manner during the debridement procedure and transported in thioglycollate broth to the microbiology laboratory. The tissue cultures were plated and incubated at 35 °C both aerobic and anaerobic on 5% sheep blood, chocolate and MacConkey agar plates, and in thioglycollate broth for 14 days or until broth turned turbid. Subcultures were done on the same primary plates. All microorganisms were routinely identified with MALDI-TOF (Bruker Daltronics, Bremen, Germany).

### Outcome

A successful outcome was defined as the absence of clinical and/or laboratory signs of infection at 1 year follow-up. Patients who required removal of the prosthesis for infectious reasons or received chronic suppressive antibiotics within the follow-up period of 1 year were considered as treatment failure. Repeated DAIR was not considered as treatment failure, and the monitoring period for treatment failure began after the last debridement procedure of the suspected early PJI episode.

### Statistical analysis

The assumption of normality was checked by visual inspection of the data using histograms and box-plots. If continuous data were normally distributed, mean and standard deviation (SD) are given, when variables were not normally distributed, median and interquartile ranges (ICR) are displayed. Patients' characteristics, preoperative laboratory results, microbiology results, and surgical and antimicrobial treatment are summarized using descriptive statistics. Differences in demographic, micro-organism, and treatment characteristics between patients with a successful and unsuccessful outcome after DAIR were analyzed using independent t-test or Mann-Whitney U-test for continuous variables and Pearson's Chi-squire test or Fisher's Exact test for categorical data. Subsequently, variables with a p-value equal or less than 0.20 were included in the logistic regression analysis to predict statistically significant correlation with outcome at 1 year follow-up.

To determine cumulative probability of infection-free implant survival a Kaplan-Meier analysis with 95% confidence intervals was used. The occurrence of infection during follow-up was used as endpoint. Patients who had their implant and no signs of infection at the end of the study period or died during the study period were censored. All statistical analyses were done using IBM SPSS statistics version 20.0. A *p*-value of less than 0.05 was considered to be statistically significant.

## Results

### Study population, patient characteristics, and survival

**Figure 1:**
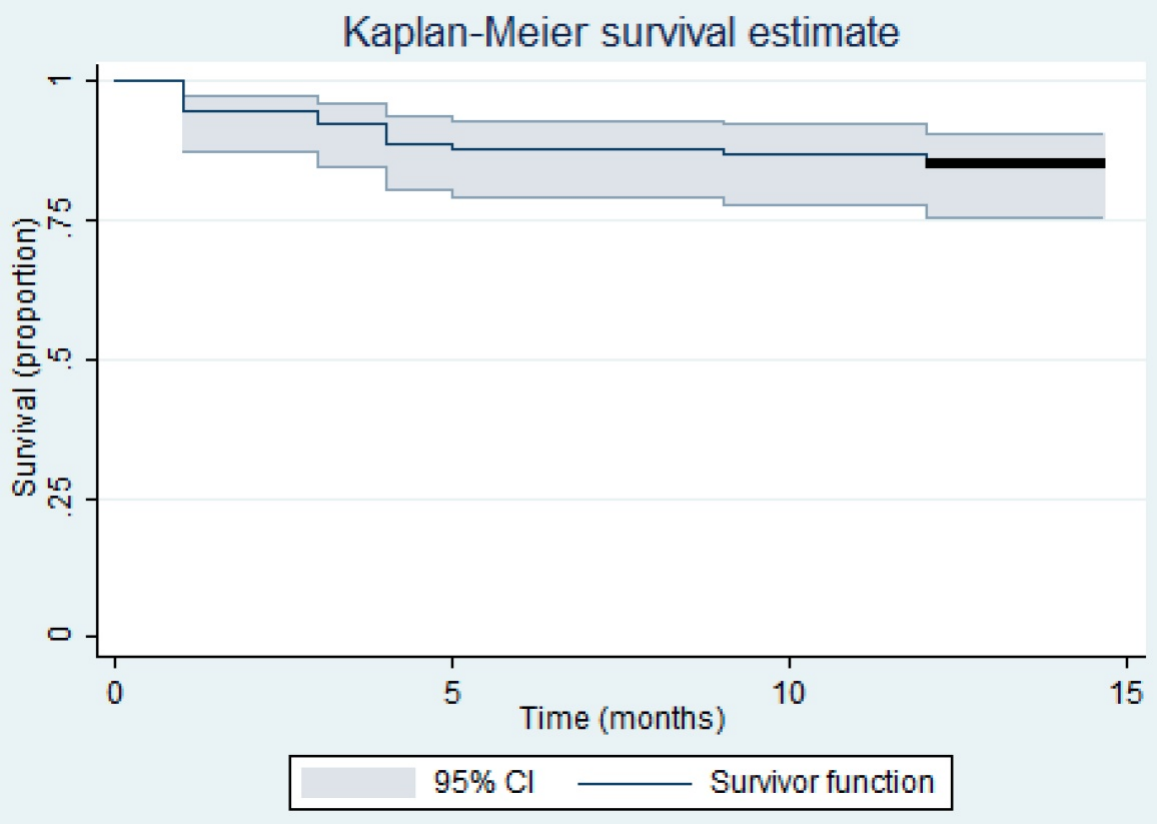
Kaplan-Meier infection-free survival curve of 91 patients treated with debridement, antibiotics and implant retention (DAIR). Censored data (vertical spikes) and 95% confidence interval (vertical bands) are also shown.

A total of 96 consecutive cases were reviewed of which 5 were excluded because follow-up after DAIR was less than 1 year. All intraoperative culture results were available in the remaining group of patients. A total of 91 patients were included in the analyses, consisting of 40 patients with a primary TKA, and 51 patients with a primary THA. Of these 91 patients 77 were free of infection without resection arthroplasty or use of suppressive antibiotics at 1 year follow-up: a success rate of 85% (95%CI 78-91) (Figure 1). No patients died during the follow-up period of 1 year. Within 1 year follow-up a 2-stage revision was performed in 8 patients, 2 patients underwent an above-the-knee amputation because of failure of previous treatment, 3 patients were ultimately treated with a permanent extraction of the THA, and 1 patient was treated with suppressive antibiotics. An overview of the patient characteristics and factors analyzed for the success and failure groups are summarized in Table 1.

### Factors associated with outcome

### Preoperative laboratory

In univariate analysis a CRP value above 100mg/L before DAIR was associated with failure (p=0.001) (Table 1). A higher mean ESR before DAIR procedure was also associated with failure (p=0.002, data not shown in table), this was not seen if a cut-off value of 30mm/hr was used (p=0.06) (Table 1). Logistic regression analysis showed that a CRP value above 100mg/L (odds ratio (OR) 10 (95%CI 1.5-70) was independently associated with failure.

### Microbiology

In 71 of the 91 patients treated with DAIR, cultures became positive with at least 2 cultures with the same micro-organism. All culture-negative cases were treated successfully. In 27 cases a polymicrobial PJI was present which did not fail more often than patient with a monomicrobial infection (p=0.1) (Table 1). The most common pathogens found were *Staphylococcus aureus* (n=29) and coagulase-negative staphylococci (CNS) (n=27) (Table 2). No methicillin resistant *Staphylococcus aureus* (MRSA) are present. The most common type of CNS were methicillin resistant (20 of 27). A higher failure rate was seen if an early PJI was caused by *Enterococcus faecalis* (p=0.04)*.* Taking into account the antibiotic resistance pattern of the isolated microorganism from intraoperative taken cultures during DAIR, 5 of 14 patients who failed after DAIR had a multiresistant microorganism (CNS & other gram-negative microorganism) (Table 2).

### Treatment

The mean number of DAIR procedures was different in the successful (1.2, SD 0.4) and unsuccessful treated patients (1.9, SD 0.8) (p=0.000) (Table 1). Patients who underwent multiple DAIRs had a higher failure rate (p=0.000). Logistic regression analysis showed a statistically significant correlation with outcome at 1 year follow-up (p=0.04, OR 5 (95%CI 1.1-23).

In 40 patients a replacement of exchangeable components took place during DAIR. No difference in success rate was seen between patients with or without the replacement of exchangeable components (p=0.6). All patients with positive cultures received prolonged antibiotics up to 15 weeks. All patients with a staphylococci infection received additional rifampicin.

## Discussion

The success rate of DAIR after primary joint arthroplasty with a high suspicion of an early PJI in our cohort (n=91) was 85%, including some patients with multiple DAIR procedures. Factors associated with treatment failure were a high CRP level (>100), multiple DAIR procedures (≥2), and an *Enterococcus faecalis* as causative microorganism.

The success rate found in this study fits within the range of previous published infection free survival rates (57-89%) 9-14. It is also in line with the best predicted probability of success of 80% after DAIR procedure with a follow-up of 1 year in the prediction model of Buller et al (2012)^15^. The relative high success rate can partly be explained by considering subsequent DAIR procedures as treatment instead as failure. In our cohort, 24 of 91 (26%) patients underwent multiple DAIR procedures of which 14 (56%) had a successful outcome. Furthermore, in a fifth of our cases tissue cultures taken during debridement remained negative. All these patients had no recurrent infection 1 year after DAIR, which suggests no significant harm is done in overtreatment in case of a suspected early PJI. If there is a suspicion of an early PJI and the appropriate minimal conditions are met (short duration of symptoms in a stable and well-fixed prosthesis with sound soft tissues and no sinus tract) DAIR is the appointed treatment to eliminate an early infection in the vast majority of patients.

Fifteen percent of our cohort failed within a year after DAIR. Previous studies identify multiple factors associated with failure after DAIR in early PJI. These factors can be grouped in preoperative available values (patient-related, symptom-related, laboratory parameters), culture/microorganism related factors, and treatment associated data. Despite taking into account patient comorbidities we did not observe a higher rate of failure in patients with American Society of Anesthesiologists (ASA) classification ≥2 16, body mass index (BMI) ≥30 17, diabetes mellitus 14, or rheumatoid arthritis 9. In addition, we did not take into account chronic renal failure and liver cirrhosis which both have been described as predictors to identify patients with a higher risk of failure 18. Other commonly mentioned preoperative values associated with failure after DAIR are serum inflammatory markers and duration of symptoms before debridement. We report a higher failure rate in patients with a high preoperative rate of CRP and ESR, which is in line with previous studies 9, 15, 18, 19. Less frequently, a WBC count of >10 (x10^9^/L) has been identified with a higher failure rate 13, 18. We did not confirm this finding. One could argue that the level of preoperative serum inflammatory markers indicates the severity of infection. Another preoperative parameter that may indicate if a suspected infection is difficult to treat, is the duration of symptoms. Earlier studies note a correlation between a longer duration of symptoms and failure after DAIR 9, 15 and a slightly better outcome if there is less time between the appearance of symptoms and debridement 20. In our study the duration of symptoms was not included due to incomplete hospital records. Therefore, we only recorded the time between the index surgery and the first debridement, which, similar to an earlier study, did not correlate with treatment failure 15.

Previous studies have outlined a higher failure rate after DAIR if infection is caused by certain microorganisms, in particularly staphylococci 9, 11, 15, 19, 21, resistant gram-negative microorganisms 22, and *Enterococcus* sp. 23-25. We only found a worse outcome in infections caused by *Enterococcus faecalis*. Taking into account the number of causative microorganisms we could not confirm the finding of previous studies indicating infections caused by multiple microorganisms tend to do worse 16, 18, 19, 26.

Treatment success is influenced by how aggressive the microorganism is treated with debridement and antibiotics. The use of ineffective empiric antibiotics significantly increases the risk of treatment failure 13, 14, 16, 22. This indicates the importance of the initial antibiotics given after debridement, based on local susceptibility data in addition to removal of inoculum and biofilm. If the microorganism is not susceptible to the antibiotic the infection is likely to flare up inducing the need for multiple debridement procedures. In our cohort multiple debridement procedures were associated with treatment failure, which is in line with previous studies 19, 22. Multiple debridement procedures can also contribute to treatment failure due to the risk of joint and wound contamination during the procedure although the results in our cohort illustrate that about 50% of patients with a high suspicion of an early PJI treated with multiple debridement procedures were treated successfully.

Our study has some limitations. First, the retrospective nature and therefore the use of data not primarily intended for research resulted in missing data. Patients were only included in the analyses if culture results were present and if a minimum follow-up of 1 year after DAIR was reached which may have contributed to selection bias and given an over- or underestimation of the success rates calculated. Furthermore, in this single center study we included a relative small number of patients, including patients suspected for an early PJIs of hip and knee resulting in a heterogeneous group. This could have contributed to a lack of power and therefore a lack in demonstrating differences between patients with a successful and failed DAIR treatment.

In conclusion, we found a success rate of DAIR after primary joint with a high suspicion of an early PJI of 85%, including some patients with multiple debridement procedures. If there is a suspicion of an early PJI DAIR is the appointed treatment. Since culture-negative DAIRs were not related to any complications during follow-up, overtreatment of a suspected PJI seems to do no significant harm with respect to implant failure. Significantly elevated preoperative serum inflammatory parameters may indicate difficult-to-treat, fulminant infections. The need for more than 2 debridement procedures is not contributive. The winning team in the treatment in suspecting an early PJI and prevention of implant failure is the use of an adequate and timely debridement technique and appropriate empiric antibiotics.

## Figures and Tables

**Table 1:** Characteristics and variables of 91 patients treated with debridement, antibiotics, and implant retention (DAIR). Results are shown for the total group, the group with successful treatment, and the group with failure of treatment.

Characteristic	Total group (n=91)	Success (n=77)	Failure (n=14)	*p*-value (univariate)	p-value (adjusted)	Odds ratio [95%CI]
**Age ^a^**	64 (12)	64 (12)	64 (10)	0.9		
**Sex, F/M**	45/46	38/39	7/7	1.0		
**Comorbidities**						
ASA classification: 1/2/3	12/63/16	11/53/13	1/10/3	0.7		
BMI ^a^	31 (7)	31 (7)	33 (5)	0.3		
Diabetes mellitus	8	5	3	0.1	0.3	
Rheumatoid arthritis	8	8	-	0.4		
Malignancy	12	10	2	1.0		
Previous PJI other joint	4	2	2	0.1	0.9	
Use of immunosuppressive agents	7	7	-	0.6		
**Joint localization**				0.6		
Knee	40	33	7			
Cemented/non-cemented/hybrid	40/0/0	33/0/0	7/0/0			
Hip	51	44	7			
Cemented/non-cemented/hybrid	18/29/4	18/24/2	0/5/2			
**Reason for joint arthroplasty**				0.5		
Primary arthrosis	68	59	9			
Posttraumatic arthrosis	8	7	1			
Rheumatoid arthritis	4	4	0			
Avascular necrosis	3	2	1			
Dysplasia	3	2	1			
Morbus Perthes	2	1	1			
Postinfectious	1	1	0			
Missing data	2	1	1			
**Diagnosis of PJI**						
Median time from implant to debridement in days ^b^	16 (12-23)	13 (11-24)	16 (13-24)	0.7		
Fever (>38,5°C)	13	10	3	0.4		
Persistent wound leakage	81	68	13	1.0		
CRP >100 mg/L	19 of 91	11 of 77	8 of 14	0.001	0.02	10 [1.5-70]
ESR >30 mm/hr	62 of 90	49 of 76	13 of 14	0.06	0.4	
Missing ESR data	1	1				
WBC >10.0 cells/µL	40 of 90	34 of 76	6 of 14	0.9		
Missing WBC data	1	1				
**Surgical treatment**						
Mean number of procedures ^a^	1.3 (0.6)	1.2 (0.4)	1.9 (0.8)	0.000		
Single/multiple DAIR procedure	67/24	63/14	4/10	0.000	0.04	5 [1.1-23]
Replacement of exchangeable components	40	33	7	0.6		
Gentamycin beads used	1	1	-	1.0		
**Microbiological diagnostics**						
Culture negative/positive	20/71	20/57	0/14	0.03	1.0	
Mono/polymicrobial	44/27	35/22	9/5	0.1	0.1	
Antibiotic, duration in weeks ^b^	13 (7-13)	13 (7-13)	12 (7-15)	0.8		
Missing data	3	3				
Empiric antibiotic treatment (adequate/inadequate)	63/28	54/23	9/5	0.8		

ASA: American Society of Anesthesiologists; BMI: Body Mass Index (kg/m^2^); PJI: prosthetic joint infection; CRP: C-reactive protein; ESR: erythrocyte sedimentation rate; WBC: white blood cell; DAIR: debridement, antibiotics, and implant retention. ^a^ Values are mean (standard deviation). ^b^ Values are median (interquartile ranges).

**Table 2:** Micro-organisms identified in 91 patients treated with debridement, antibiotics, and implant retention (DAIR) (including polymicrobial infections). Result are shown for the total group, the group with successful treatment, and the group with failure of treatment.

Microorganisms	Total (n=91)	Success (n=77)	Failure (n=14)	p-value	
**Culture negative**	20	20	-	0.03	
**Gram-positive** (number of resistant microorganisms)	67	53	14	0.02	
*Staphylococcus aureus*	29 (0)	22 (0)	7 (0)	0.1	
*CNS*	27 (20)	22 (17)	5 (3)	0.8	
*Streptococcus spp.*	8 (0)	8 (0)	-	0.4	
*Enterococcus faecalis*	10 (0)	6 (0)	4 (0)	0.04	
*Corynebacterium spp.*	8 (2)	6 (2)	2 (0)	0.6	
*Propionibacterium spp.*	6 (0)	6 (0)	-	0.6	
*Other^┼^*	2 (0)	2 (0)	-	1.0	
**Gram-negative** (number of resistant microorganisms)	15	11	4	0.2	
*Escheria coli*	5 (0)	3 (0)	2(0)	0.2	
*Proteus mirabilis*	4 (1)	4 (1)	-	1.0	
*Enterobacter spp.*	2 (2)	2 (2)	-	1.0	
*Pseudomonas aeruginosa*	2 (0)	1 (1)	1 (0)	0.3	
*Other^ǂ^*	5 (3)	2 (1)	3 (2)	0.03	

## Abbreviations

DAIR: debridement, antibiotics, implant retention
PJI: prosthetic joint infection
TKA: total knee arthroplasty
THA: total hip arthroplasty
CRP: C-reactive protein
ESR: erythrocyte sedimentation rate
WBC: white blood cell
CNS: coagulase-negative staphylococci
MRSA: methicillin resistant *Staphylococcus aureus*
BMI: body mass index
ASA: American Society of Anesthesiologists
